# Modification of Negative Pressure Wound Therapy and Mesh-Mediated Fascial Traction for Open Abdomen Treatment

**DOI:** 10.7759/cureus.79153

**Published:** 2025-02-17

**Authors:** Rastislav Burda, Theodoz Molčányi, Marek Molčányi, Ildiko Morochovičová, Ivan Kováč

**Affiliations:** 1 Department of Trauma Surgery, Pavol Jozef Safarik University, Kosice, SVK; 2 Department of Trauma Surgery, Hospital of L. Pasteur, Kosice, SVK; 3 Institute of Neurophysiology, University of Cologne, Cologne, DEU; 4 Department of Physiotherapy, Faculty of Medicine, Pavol Jozef Safarik University, Kosice, SVK; 5 2nd Department of Surgery, Pavol Jozef Safarik University, Kosice, SVK

**Keywords:** acute abdominal compartment syndrome, delayed closure, mesh-mediated fascial traction, negative-pressure wound therapy, open abdomen surgery

## Abstract

The introduction of the open abdomen technique for laparostomies has presented new problems, including the method of temporary coverage and the primary and delayed closure of the laparostomy. Numerous techniques for the delayed closure of a laparostomy have been described in the literature, but closure of a laparostomy with a colostomy present is a more technically challenging situation. The combination of negative pressure wound therapy and mesh-mediated fascial traction is now considered the method of choice.

This paper presents a modification of the negative pressure wound therapy and mesh-mediated fascial traction techniques, by which laparostomy closure can be easily and quickly achieved by applying mesh as a whole and applying traction on the excess part. The traction on different parts of the mesh can be easily adjusted to avoid colostomy compression.

## Introduction

Leaving an open abdomen following an emergency laparostomy is a well-known resuscitative maneuver for life-threatening abdominal conditions such as trauma, sepsis, acute abdominal compartment syndrome, a ruptured abdominal aortic aneurysm, intestinal ischemia, and other conditions [1].

Despite its advantages, the open abdomen approach is associated with significant morbidities, including intestinal fistulation, bleeding, intestinal failure, and high overall mortality (28.2%) [2]. The open abdomen technique has led to the successful treatment of acute abdominal compartment syndrome, but it has also presented problems, such as the method of temporary coverage and the primary and delayed closure of the laparostomy.

The situation is even more complicated in the presence of an intestinal suture or colostomy [3,4]. Not all cases of open abdomen treatment can be treated using conventional techniques, as in some cases it is necessary to modify the available methodologies according to the patient's requirements.

## Case presentation

The patient (a 56-year-old man) fell from a horse during rodeo practice, and then the horse fell onto the patient’s abdomen. Initial abdominal computed tomography (CT) scans revealed a small amount of free fluid in the Douglas space and a contusion of the mesentery. The patient complained of non-specific abdominal pain, the abdomen was tense, and there were no signs of peritoneal irritation on examination. The patient's abdominal pain persisted; it was localized in the lower abdomen. On the second day, the local finding worsened, the patient had signs of peritoneal irritation, and the finding on the CT examination was also changed. A repeated CT scan two days later revealed an increase in free fluid in the small pelvis. The colon had a narrowed wall instead of the free fluid, without obvious pneumoperitoneum. Based on the CT examination, a suspicion of colon rupture was pronounced.

**Figure 1:**
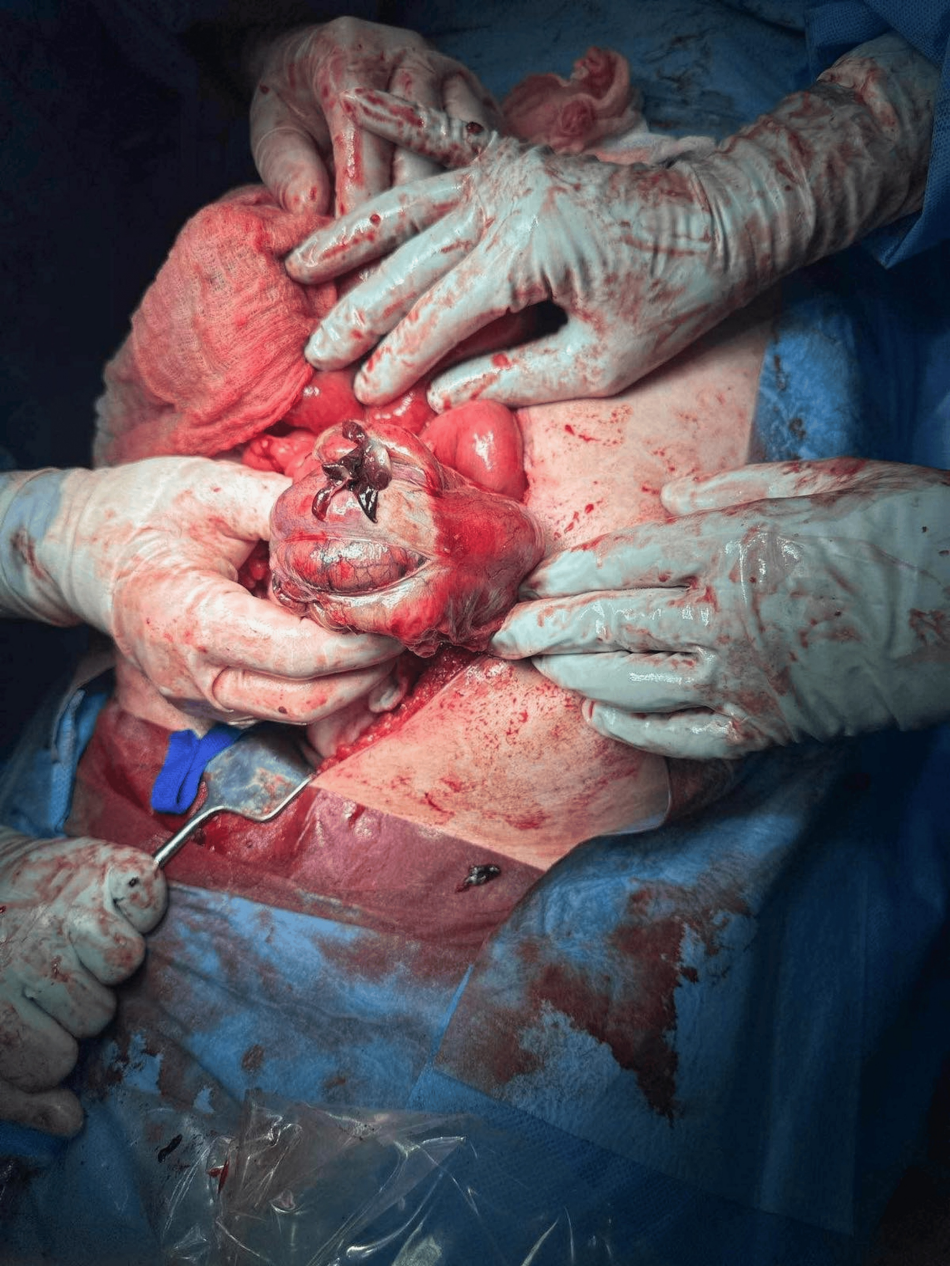
Initial view of a cecal rupture with the avital intestinal wall clearly visible around the rupture.

Worsening local abdominal findings in the correlative with CT results were an indication of revision using upper median laparotomy. During the procedure rupture of the sigmoid colon with localized peritonitis was identified (Figure 1).

Therefore, resection of the sigmoid colon using Hartmann’s procedure - the closure of the aboral part by stapler, resection of the damaged and inflamed part of the colon, and the placement of a colostomy above the site of the colon damage - was the treatment of choice.

**Figure 2:**
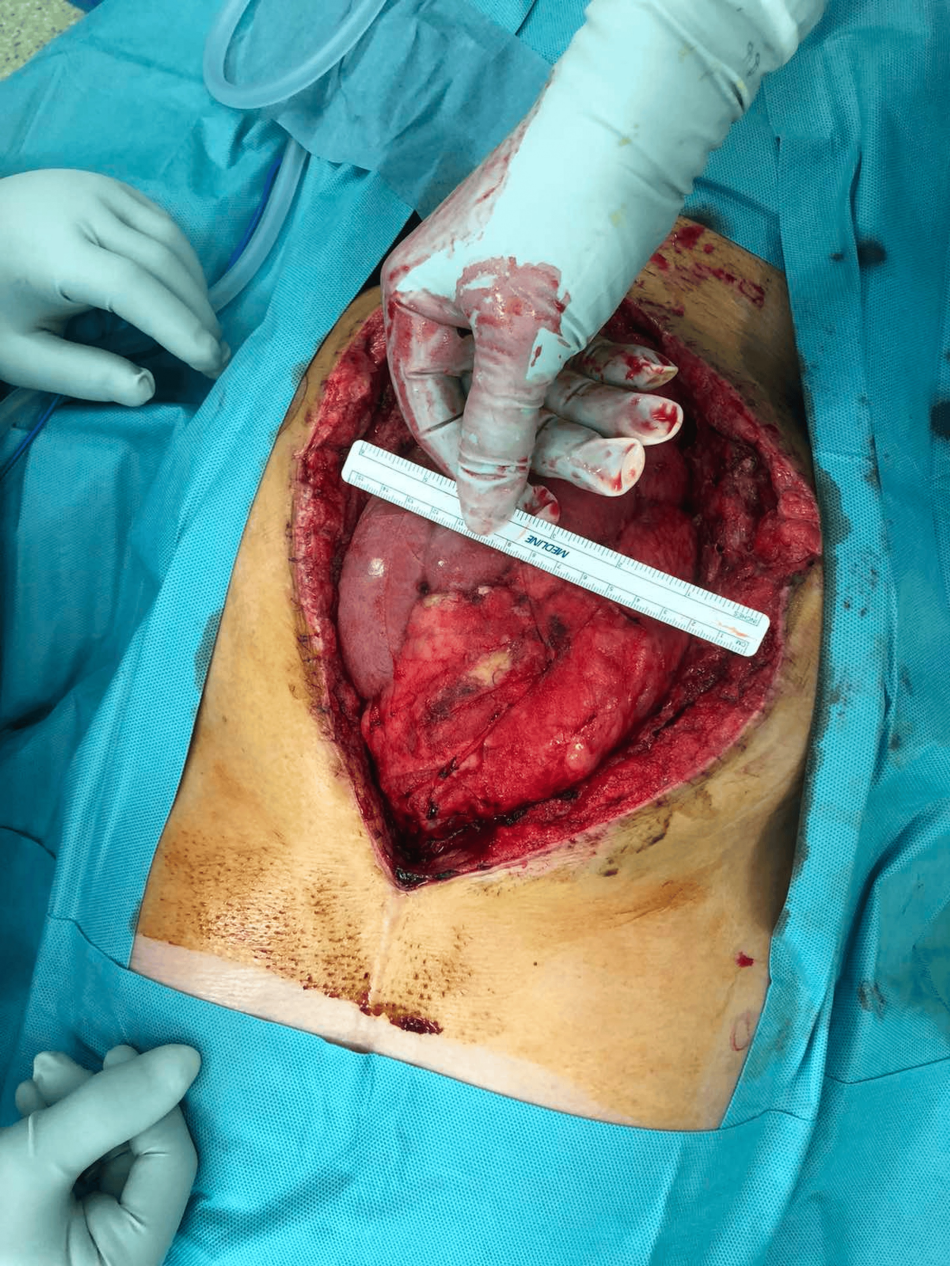
Size of the laparostomy is clearly visible in the image.

Postoperatively, a hematoma developed in the wound, and dehiscence of the surgical wound occurred. This led to a surgical revision in which the hematoma was evacuated, and due to the presence of massive intestinal edema, primary laparostomy closure was contraindicated because of the high risk of developing abdominal compartment syndrome, so the open abdomen technique was chosen (Figure 2) with the application of temporary negative pressure wound therapy (NPWT). Since there was no significant reduction in bowel swelling after several days, primary closure was not possible. Moreover, excessive pressure could lead to compression of the colostomy.

**Figure 3:**
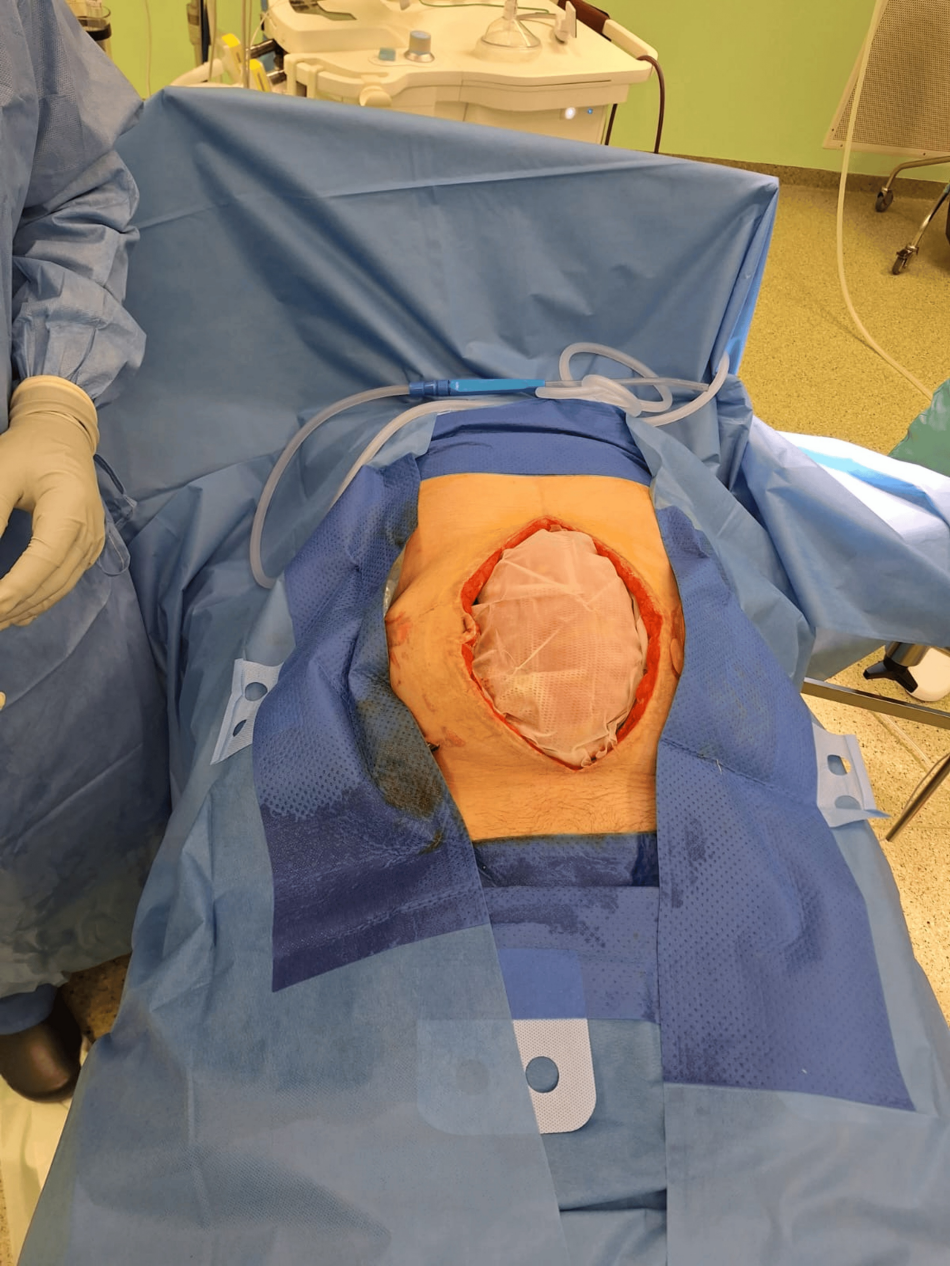
Insertion of mesh into the open abdomen using the inlay technique. The center of the mesh is loose to avoid pulling on the abdominal wall.

For the above reasons, a modified mesh-mediated fascial traction (MMFT) technique for the delayed primary closure of the open abdomen was applied. The laparostomy showed no signs of infection, and repeated microbiological smears did not demonstrate any intra-abdominal infection, so the decision was made to implant a composite polyester mesh (Symbotex™ composite mesh, Medtronic, USA) (Figure 3).

This mesh was inserted using the inlay technique and fixed using a fixation device of prosthetic material (ProTack, Medtronic, USA). There was no anti-adhesive layer placed under the mesh. The mesh was coated with a bioabsorbable collagen that minimizes soft tissue attachment. The mesh was not inserted under tension; it was loose in the center to prevent the occurrence of abdominal compartment syndrome. A sponge was placed on the mesh through the use of NPWT therapy. Before the mesh insertion, two drains were inserted in the paracolic gutters in the abdominal cavity.

**Figure 4:**
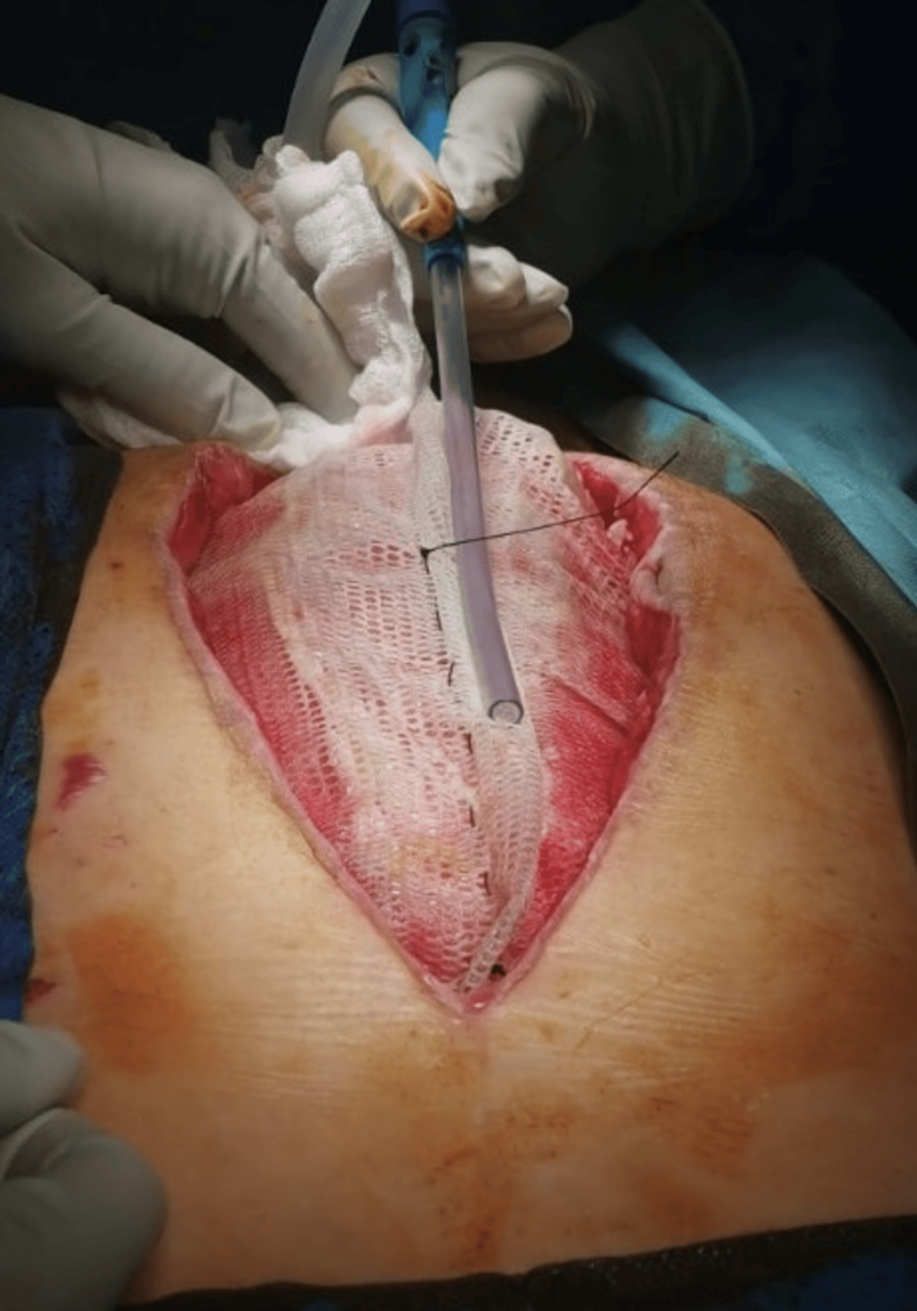
Beginning of the first plication of the mesh with a continuous suture from the center of the mesh toward the distal.

**Figure 5:**
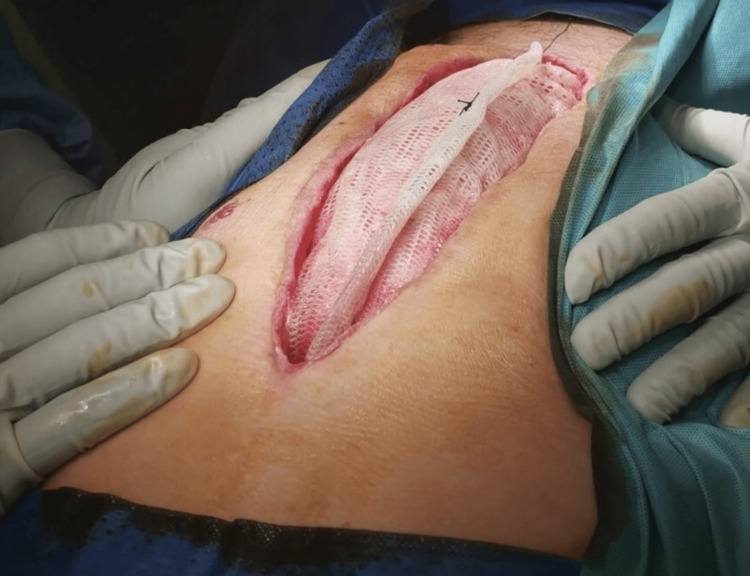
Manual massage of the lateral abdominal wall. Optimal tension of the mesh is achieved by manual massage of the lateral abdominal wall and traction on the apex of the plication.

**Figure 6:**
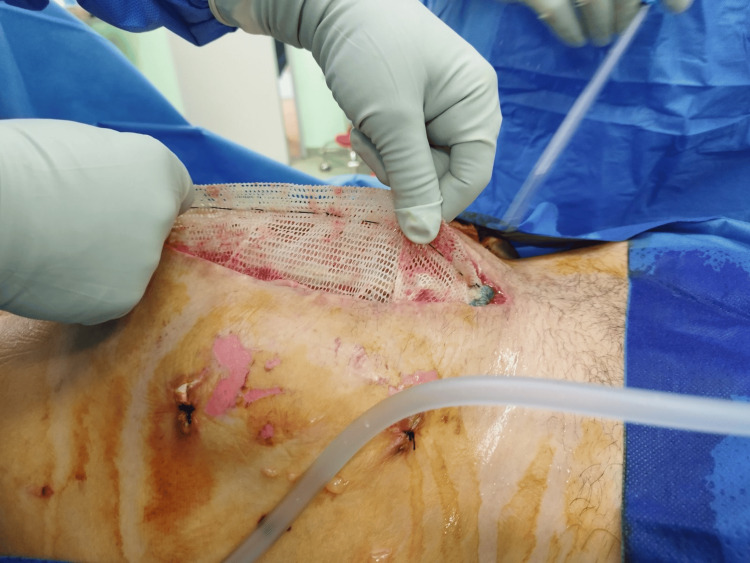
Revision after 48 hours, mesh tensioning. Revision after 48 hours, the extent of the residual defect is significantly smaller, so it is possible to carry out a second plication of the mesh.

**Figure 7:**
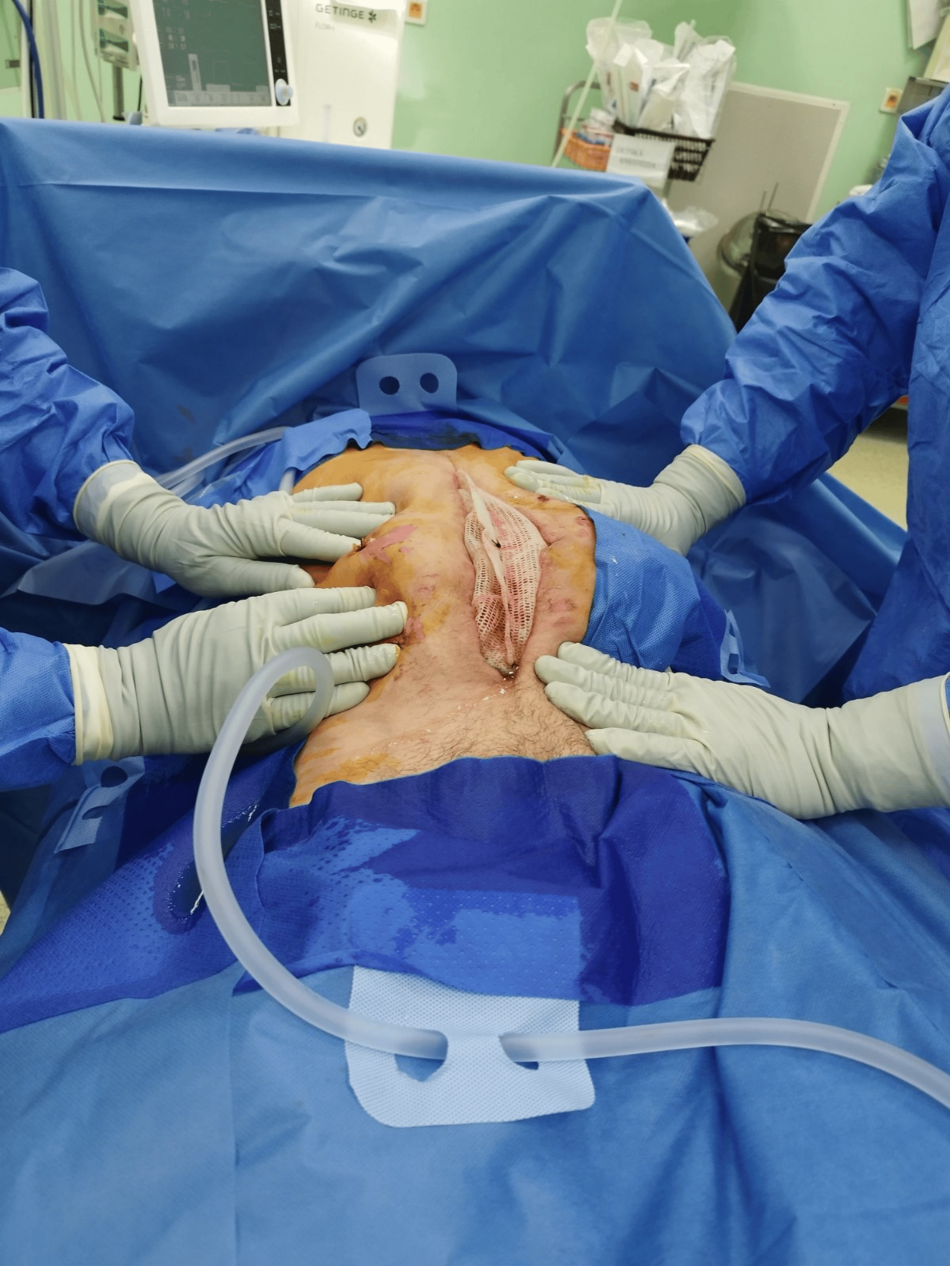
Manual medialization of the edges of the abdominal wall.

**Figure 8:**
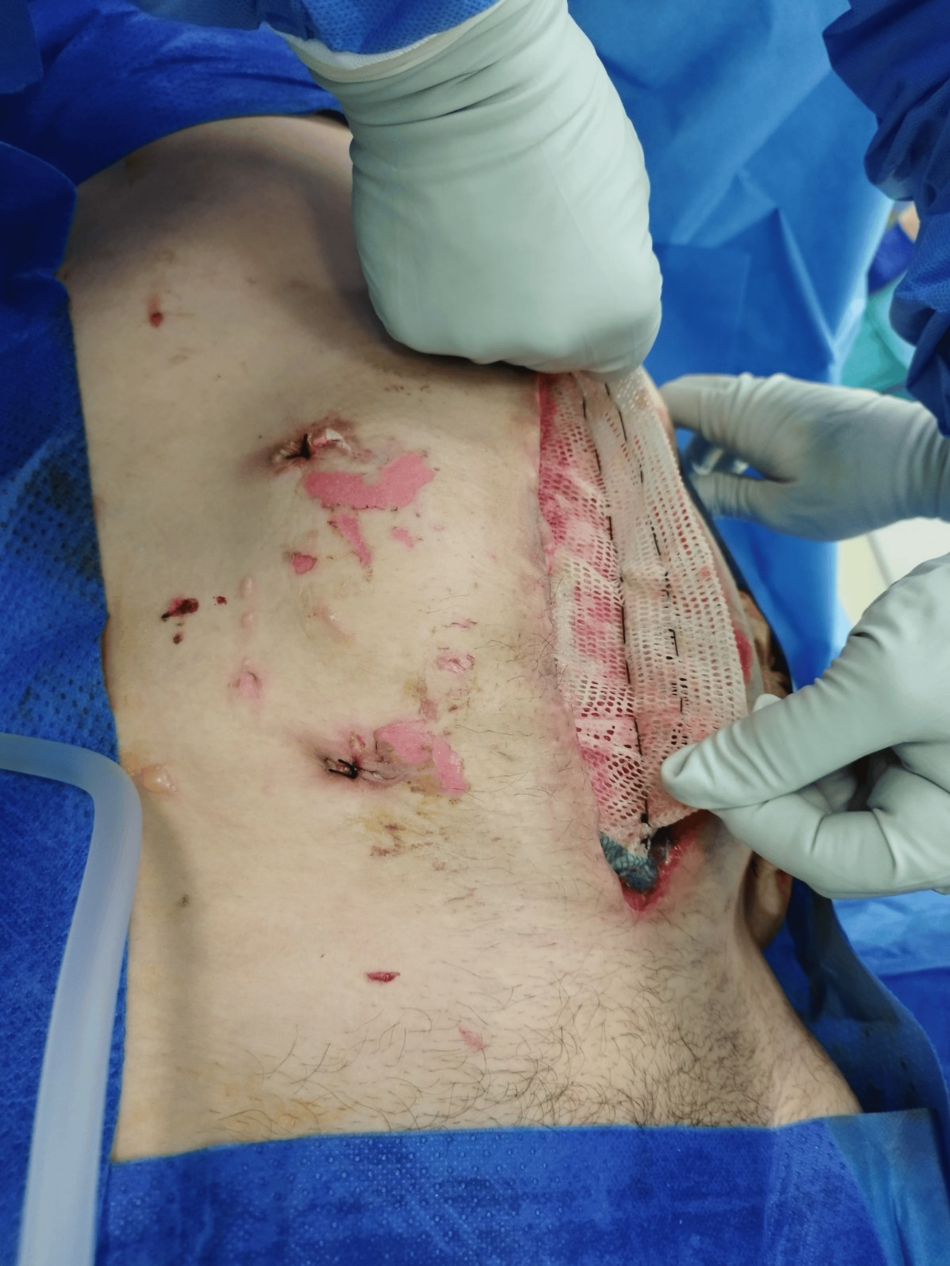
A close-up view of double-ply mesh.

**Figure 9:**
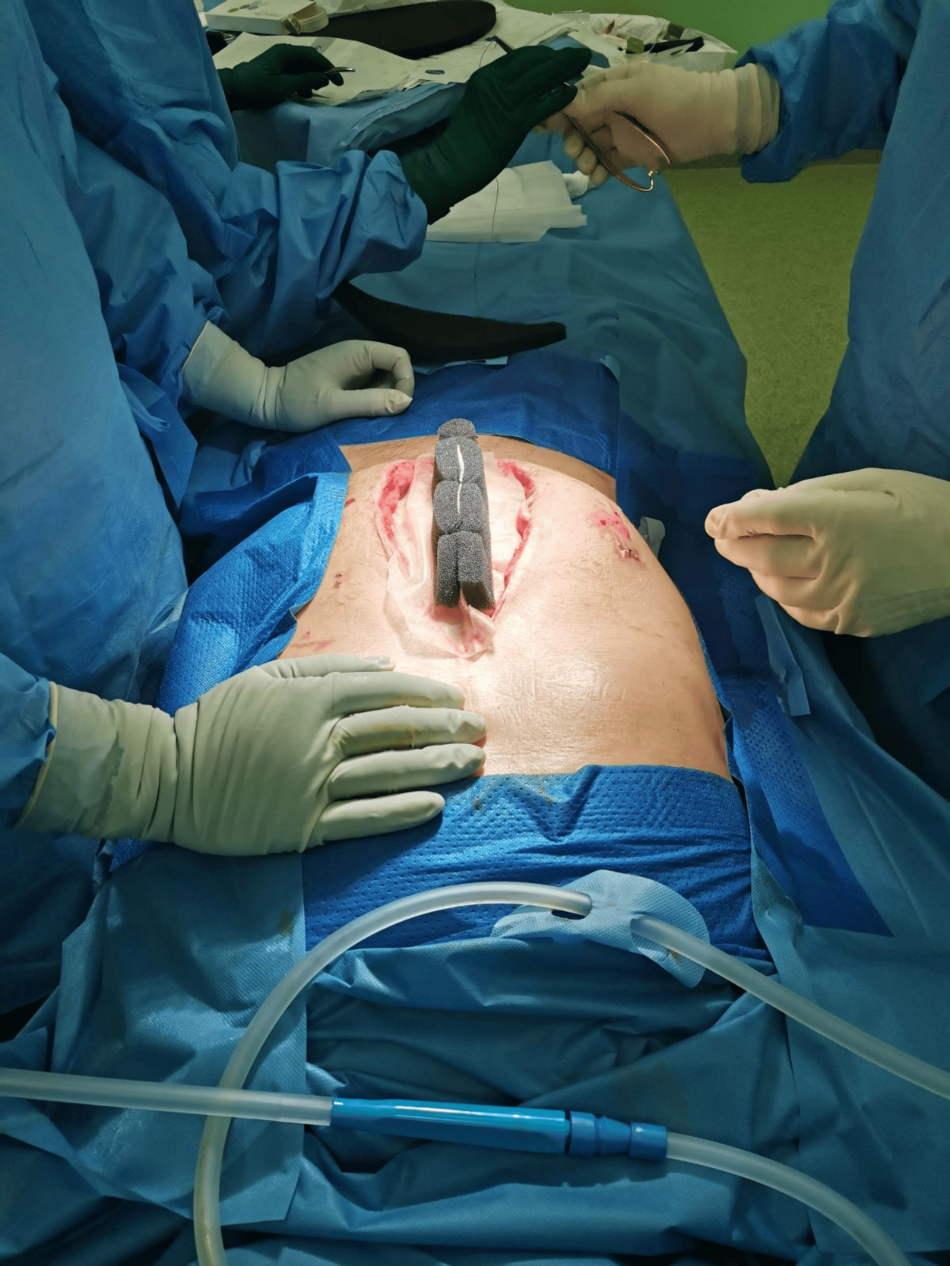
Negative pressure wound therapy application to plied mesh. The pleated mesh is lined with a sponge and then applied negative pressure wound therapy.

**Figure 10:**
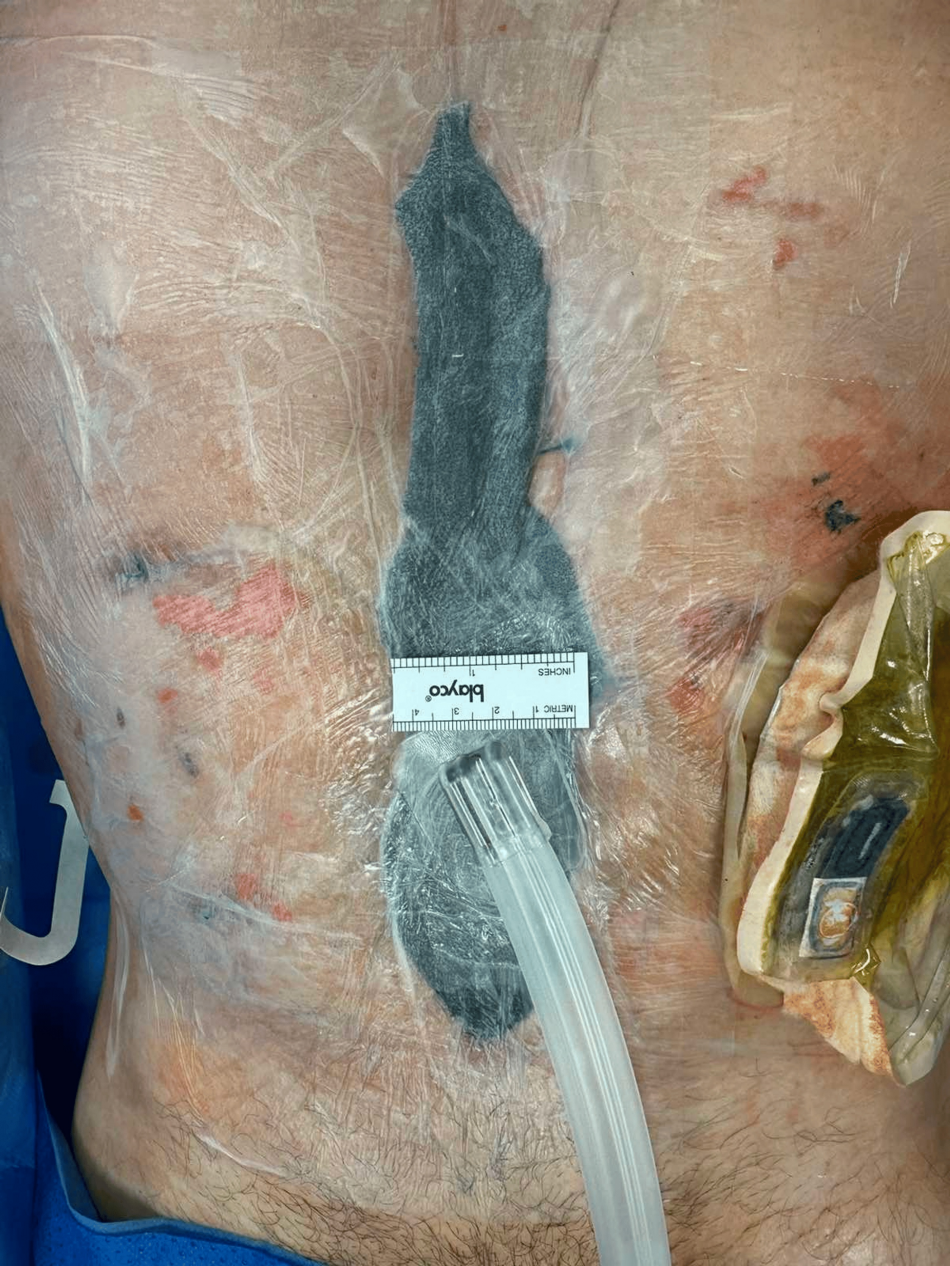
Negative pressure wound therapy application to plied mesh. The pleated mesh is lined with a sponge, then another sponge is applied to the entire residual abdominal wall defect.

**Figure 11:**
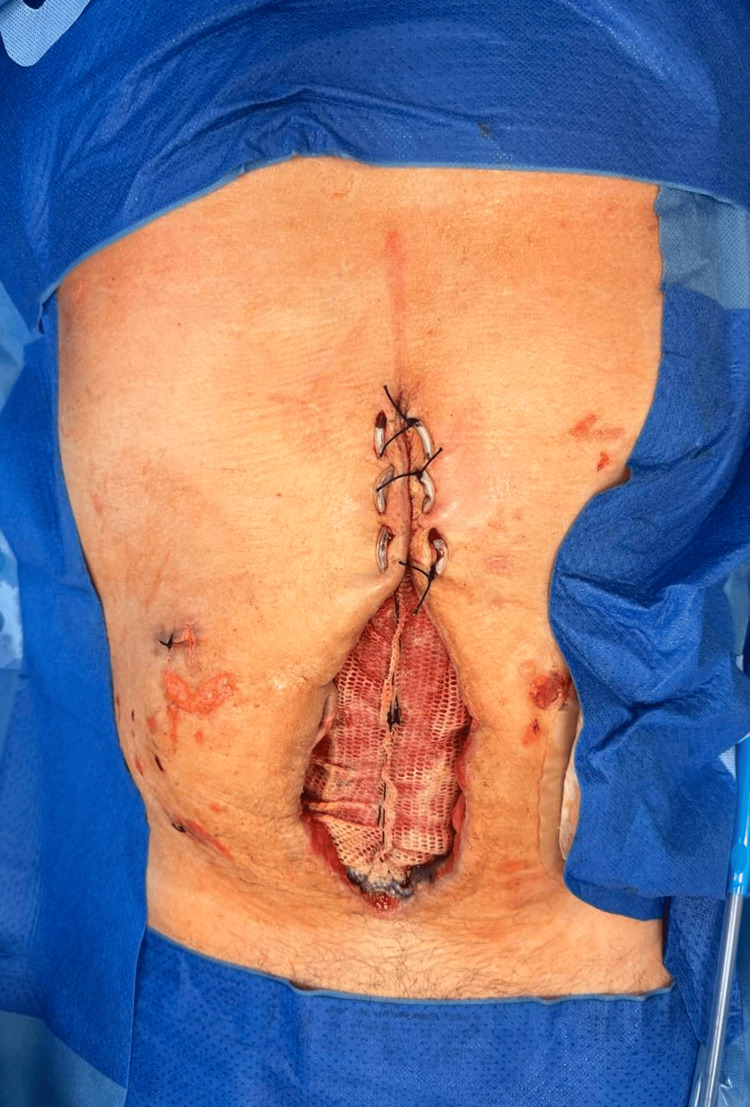
View of delayed closure of the abdominal wall. U-sutures placed at the proximal part of laparostomy.

**Figure 12:**
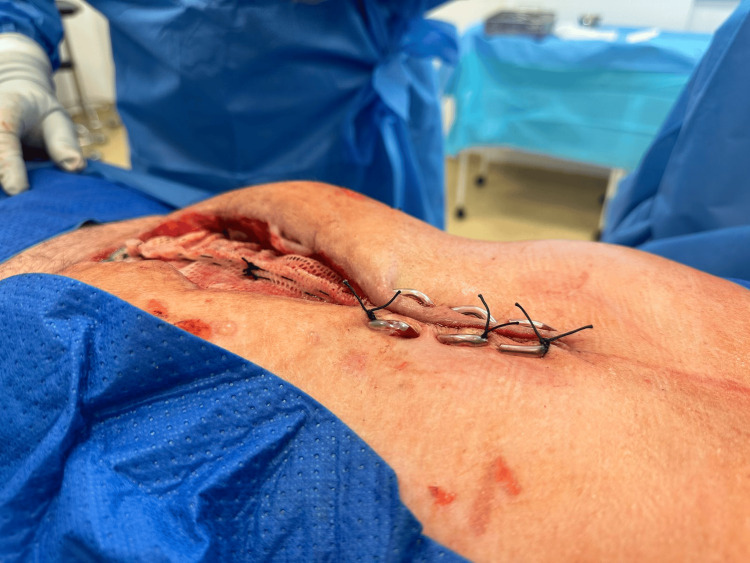
A close-up view of a partially closed laparostomy and plicated mesh.

**Figure 13:**
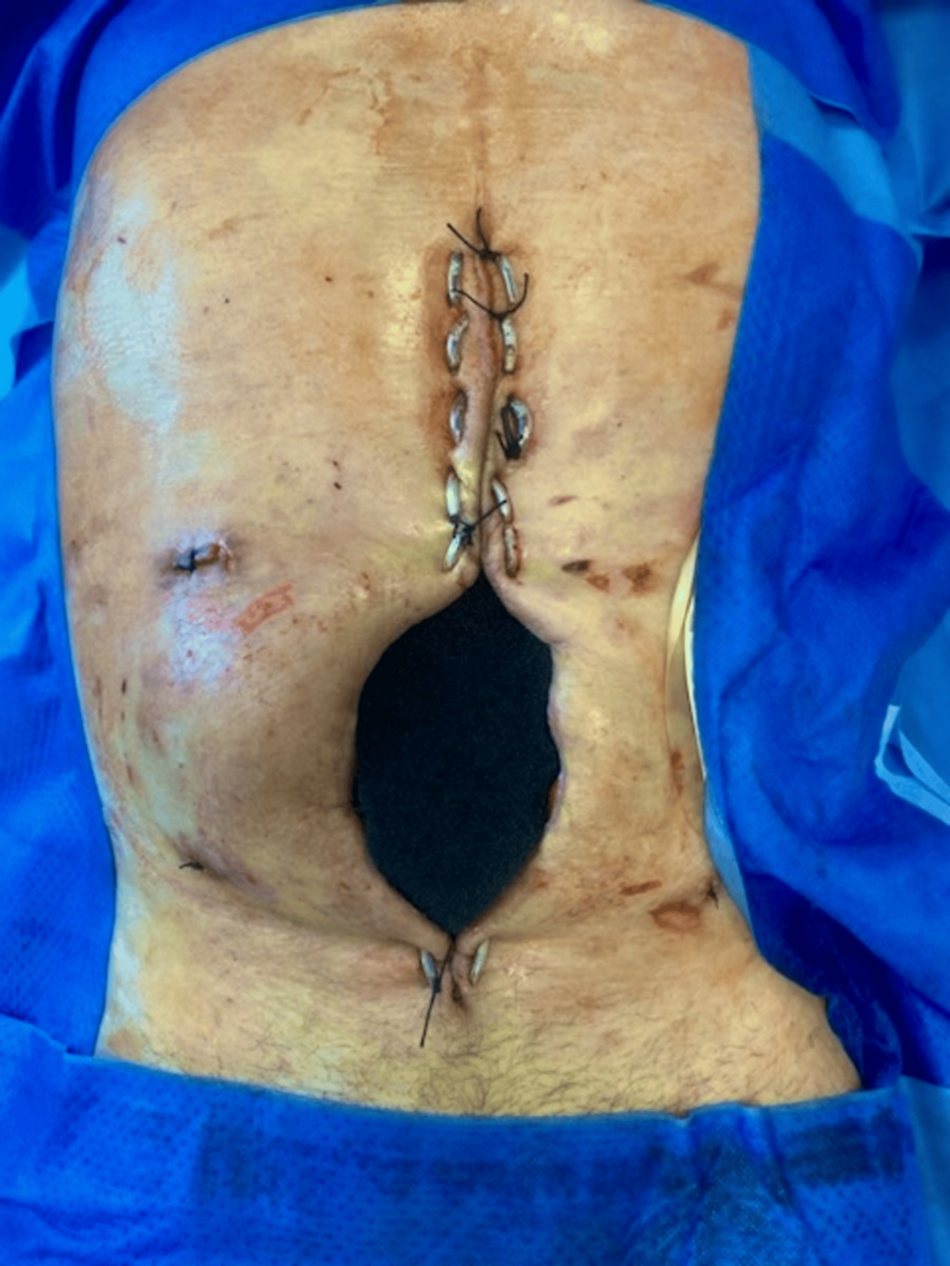
NPWT application during gradual abdominal closure. Sponge application to residual laparostomy. NPWT: negative pressure wound therapy

During subsequent dressings (two days later), the excess mesh was not resected as in the conventional technique, but a plication was created in the free center of the mesh, which was fixed with a continuous suture from the center of the mesh in both the proximal and distal directions. The continuous suture at the base of the gather was left in place. After another two days, the mesh was again pretensioned in the center. The new plication shortened the mesh diameter by 4 cm, and after the new pleating, the new continuous suture was re-introduced at the base of the gather (Figures 4-13).

A sponge and NPWT were still applied over the mesh. Since the aforementioned mesh has large holes, the application of NPWT over the mesh allowed direct suction of the fluid from the abdominal cavity. The mesh did not adhere quickly to the intestines due to the collagen film on the mesh. Additionally, U-shaped sutures were gradually applied through a rubber tube from the proximal part of the laparostomy to the distal part. The wound was closed in the above manner in three sessions. The sutures were applied to the entire thickness of the abdominal wall, allowing for gradual convergence of the abdominal wall across the entire width. The excess remnants of mesh were finally resected along the entire length of the wound and sutured with non-absorbable material under slight tension to avoid harming the colostomy.

**Figure 14:**
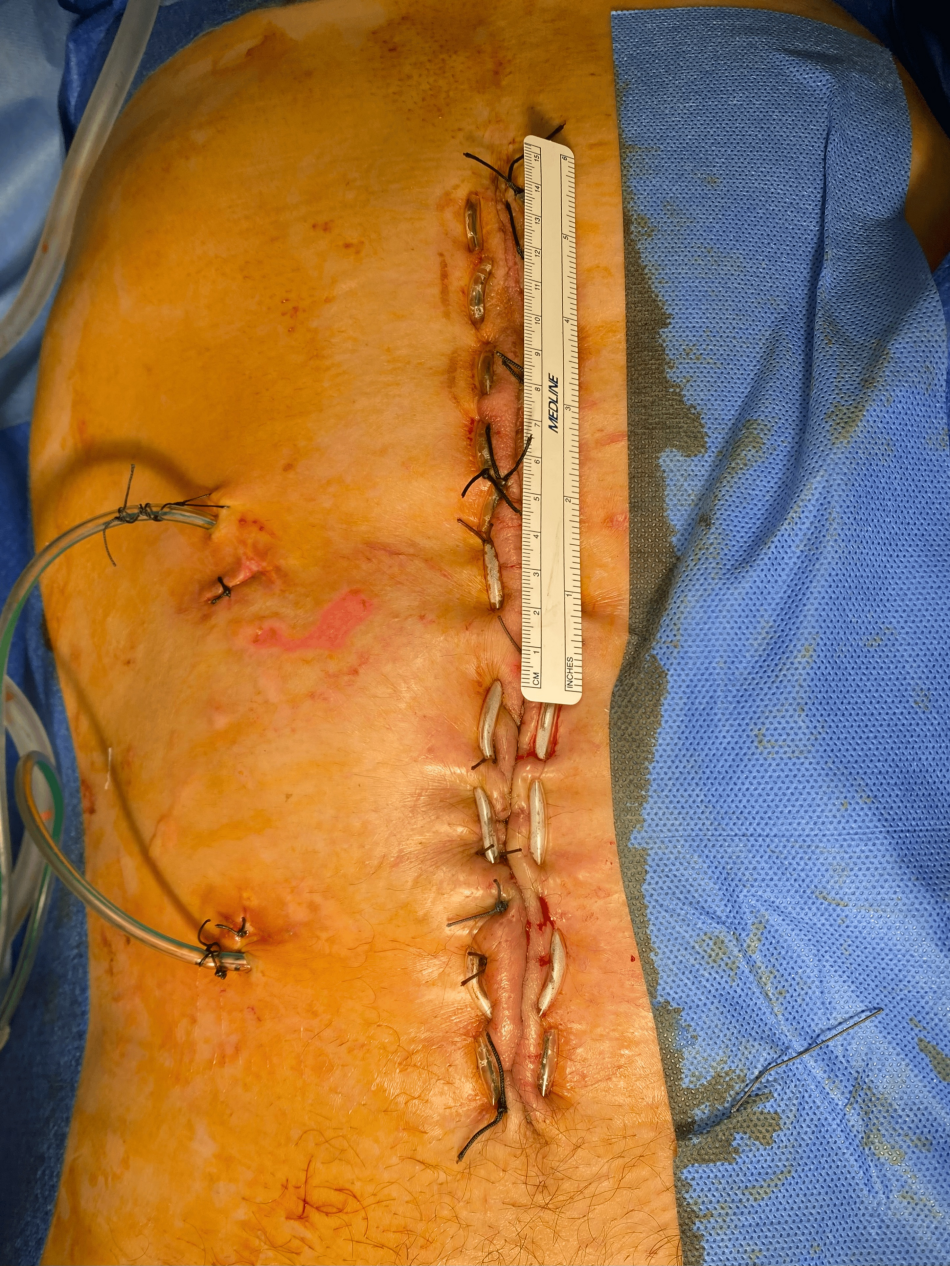
Fully closed open abdomen. Detailed view of a closed laparostomy, the abdominal wall is pulled together with U-shaped sutures across the entire wall's width.

The laparostomy was closed in the abovementioned manner after 14 days in five gradual steps, during which the patient was anesthetized. U-shaped sutures remained for eight weeks, after which they were gradually removed (Figure 14).

**Figure 15:**
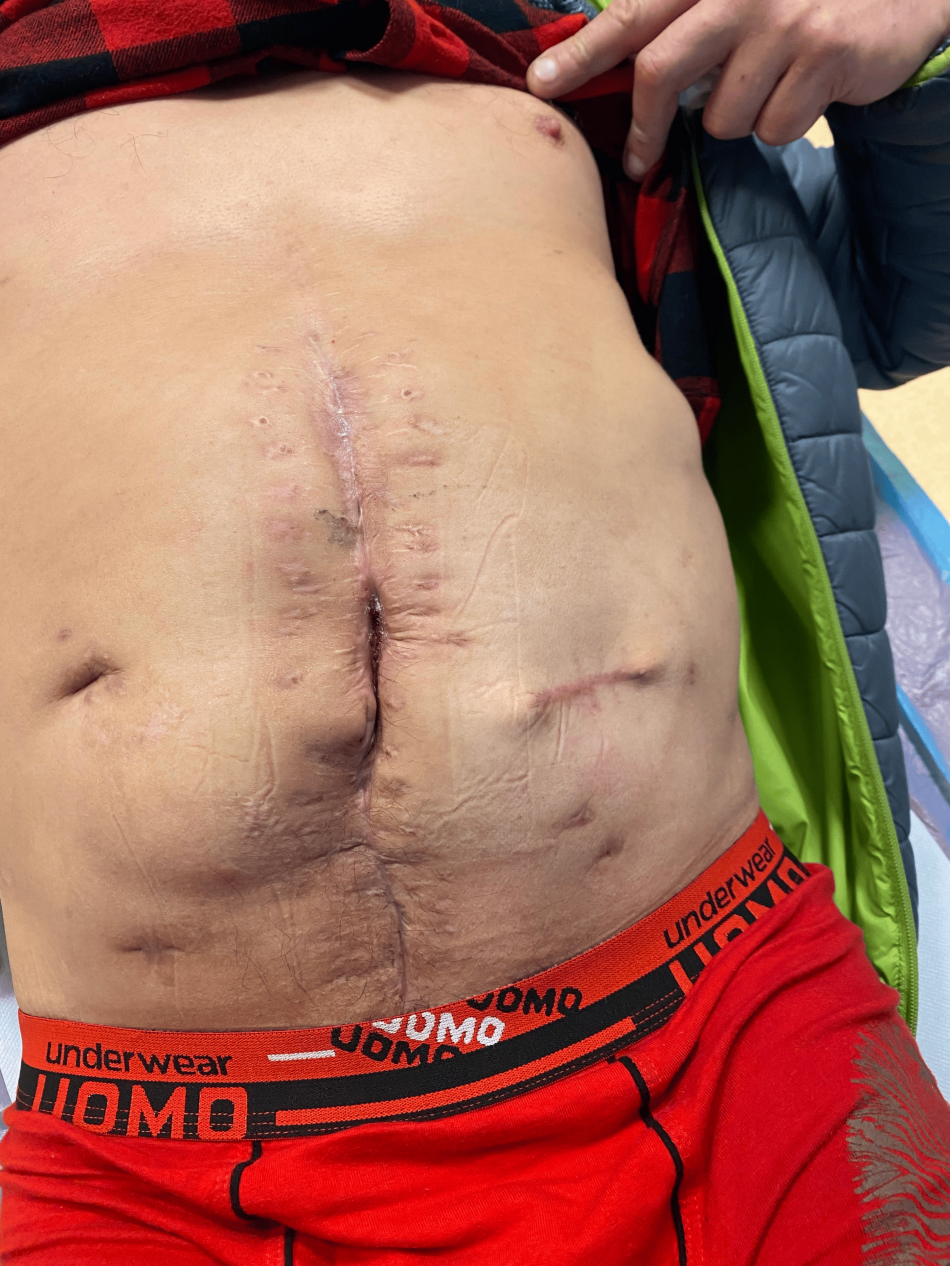
Final follow-up showing a healed abdominal wall without a hernia formation.

After six months, the colostomy was removed, and the continuity of the colon was restored without subsequent complications (Figure 15).

## Discussion

After a laparotomy, primary closure of the abdominal fascia is typically the preferred approach and the goal of treatment is to achieve early primary closure of the laparostomy, which is usually achieved in the majority of trauma patients but is much more difficult in non-trauma cases. Compared with delayed abdominal closure, early fascial closure significantly reduced mortality and complication incidence.

Patients undergoing temporary abdominal closure after non-trauma emergency laparotomy had a significantly greater risk of postoperative complications [5-7]. Temporary closure of the abdominal cavity with plastic bags, silicone sheets, absorbable and non-absorbable meshes sutured to the fascial or skin edges and NPWT do not facilitate the definitive closure of the abdominal wall [8,9].

NPWT is the most frequently used temporary abdominal closure technique; however, it is associated with a reduced rate of delayed primary closure. Closure rates after NPWT of open abdomen treatment in trauma patients have been reported to be 86%-92% [10,11]. The majority of patients were young and were treated using the damage control strategy, but some patients were older, had accompanying renal failure, and suffered from more diverse pathologies, so visceral swelling lasted longer and open abdomen treatment was required for a longer period of time. Fascial closure after three weeks is seldom possible after prolonged open abdomen therapy. Delayed primary closure may be necessary if the open abdomen cannot be closed immediately after the resolution of the acute problem.

A number of different techniques have been proposed and described for delayed definitive abdominal closure. Options include component separation, split skin grafting, and MMFT. The highest weighted fascial closure rate was found in a series describing NPWT with a continuous mesh or MMFT and dynamic retention sutures [12,13]. Skin-only closure or split-thickness skin grafting may also be used to cover the bowel and omentum, but the major drawback with these techniques is the formation of extensive ventral hernias that have to be addressed later on.

MMFT was first described by Petersson et al. in 2007 [13]. Further reports have shown fascial closure rates of 61%-100% in patients surviving open abdomen treatment, but despite the success in achieving fascial closure, incisional hernia rates in the range of 35%-62% have been reported [14,15]. The use of a visceral protection layer (VPL) is highly recommended when using NPWT on the open abdomen because it has been documented to reduce the rate of enteroatmospheric fistula in cases of peritonitis due to the perforation of a hollow viscus or anastomotic insufficiency [14]. If a stoma is present, a radial incision can be made in the VPL to accommodate this. Woven mesh is used to allow the passage of exudate to the NPWT collection device. A large-sized medium- to heavy-weight woven polypropylene mesh is recommended [1]. The combination of NPWT and MMFT gives the highest rate of fascial closure for delayed primary closure [12]. Guidelines published later also recommend the combination of NPWT and MMFT as the technique of choice [16,17].

An advantage of MMFT combined with NPWT or the Wittmann Patch technique compared to abdominal vacuum-assisted wound closure combined with partial suturing of the fascia sequentially is the possibility of cleansing the entire abdominal cavity during the period of open abdomen treatment, where the total length of the incision is accessible until the fascia is closed [11,18]. Moreover, MMFT due to the porosity of the mesh allows adequate drainage of peritoneal fluid from the abdominal cavity.

The original MMFT technique is based on circumferential suturing of the mesh to the fascia only, not to the abdominal muscles, as this can lead to muscle ischemia. The mesh is then cut in the middle, creating two edges, which can be pulled to tighten the fascia. Excess mesh is excised before tightening. The edges of the mesh are sutured under adequate tension, and foam and occlusive foil are applied to the mesh. Progressive tightening of the mesh can be performed every 48 to 72 hours, while the edges of the mesh are again trimmed and sutured under slight tension with a continuous non-absorbable suture. Definitive suturing of the fascia is only possible when the edges of the fascia can be brought closer together.

A specific situation arises in the presence of a stoma when the fascia pull must be gentle to avoid ischemia of the stoma or its mechanical strangulation. The original technique still recommends the application of a VPL under the mesh.

For this study, a modified MMFT technique was used in which the mesh was not cut in the middle but was left whole. Subsequent traction was not achieved by pulling the cut edges of the mesh together but by creating a repeated plication in the middle of the mesh, which allows for a more even distribution of tension on the abdominal wall in the presence of a stoma.

In the case of recurrence of abdominal compartment syndrome, it is sufficient to release the sutured mesh plication and increase the mesh area. In our opinion, the introduction of abdominal drains before mesh application is not counterproductive, as it allows for monitoring of the output from the abdominal cavity during and after closure of the laparostomy and does not interfere with the applied NPWT technique.

## Conclusions

A wide variety of techniques for the delayed closure of a laparostomy have been described in the literature. Closure of a laparostomy with a colostomy present is always more technically challenging. The combination of NPWT and MMFT is now considered the method of choice.

We present a modification of the NPWT and MMFT technique, where laparostomy closure can be easily and quickly achieved by applying the mesh as a whole and traction on its excess part. In addition, the traction in different parts of the mesh can be easily adjusted to avoid colostomy compression.
